# EXPLORING AI FOR DEMENTIA CARE SUPPORT IN NORTHERN ONTARIO: A LITERATURE REVIEW

**DOI:** 10.1093/geroni/igae098.4290

**Published:** 2024-12-31

**Authors:** Hom Shrestha, Lucy Shrestha

**Affiliations:** Laurentian University, Sudbury, Ontario, Canada; Laurentian University, Sudbury, Ontario, Canada

## Abstract

Dementia is the seventh leading cause of death globally, significantly impairs cognitive abilities and memory loss. Artificial intelligence (AI) has the potential to revolutionize dementia care by assisting healthcare professionals in diagnosing, managing, and treating dementia worldwide. We conducted literature review using Google Scholar, PubMed, and Laurentian libraries (OMNI), focusing on key terms such as “dementia,” “Alzheimer’s disease,” “artificial intelligence,” and “healthcare.” This review covered peer-reviewed publications from 2014 to 2023, aiming to fill gaps in this research area. The search yielded five relevant articles, identifying three core themes: AI’s impact on Alzheimer’s disease and related dementias (ADRD), early detection and diagnosis of ADRD, and AI’s role in supporting healthcare professionals, caregivers, and patients. Zhang et al. (2023) emphasized AI’s role in diagnosing Alzheimer’s disease using MRI, PET scans, biomarkers, and advanced machine learning applications. Hane et al. (2020) highlighted the use of machine learning models for assessing ADRD risk by analyzing clinical notes to prevent nursing home admissions and reduce care costs. Xie et al. (2020) explored the use of assistive AI devices by caregivers to manage ADRD, reducing patient dependence. Li et al. (2020) focused on voice-activated AI tools that help caregivers manage nutrition and reduce their workload. Gustavsson, Svanberg, and Mullersdorf (2015) examined the benefits of the interactive robotic “Justo Cat” in enhancing communication in dementia care. This review suggests conducting ethnographic participatory focused-groups with thematic analysis to inform policymakers in developing specialized educational programs, integrating advanced AI, expanding digital health and telemedicine, and launching community-driven initiatives.

